# Causes of anxiety among teachers giving face-to-face lessons after the reopening of schools during the COVID-19 pandemic: a cross-sectional study

**DOI:** 10.1186/s12889-021-11130-y

**Published:** 2021-06-02

**Authors:** Nobuyuki Wakui, Shinichiro Abe, Shunsuke Shirozu, Yuuki Yamamoto, Miho Yamamura, Yasuyo Abe, Souichi Murata, Mizue Ozawa, Takahiro Igarashi, Takahiro Yanagiya, Yoshiaki Machida, Mayumi Kikuchi

**Affiliations:** 1 Division of Applied Pharmaceutical Education and Research, Faculty of Pharmaceutical Sciences, Hoshi University, 2-4-41 Ebara, Shinagawa-ku, Tokyo, 142-8501 Japan; 2 Shinagawa Pharmaceutical Association, 2-4-2 Nakanobu, Shinagawa-ku, Tokyo, 142-0053 Japan

**Keywords:** COVID-19, Teachers, Factors of anxiety, Face-to-face classes, School reopening

## Abstract

**Background:**

Coronavirus infections are spreading rapidly worldwide, and primary and middle schools are closed in many countries. After the state of emergency was lifted in Japan, schools have reopened, and teachers are conducting face-to-face classes while maintaining safety precautions. This study aimed to assess the factors contributing to infection-related anxiety and educational anxiety among teachers conducting face-to-face classes during the COVID-19 pandemic after schools reopened.

**Methods:**

This questionnaire-based cross-sectional study was conducted with 263 primary and middle school teachers in the Shinagawa area of Tokyo (October 10–30, 2020). The questionnaire assessed the type of school (primary or middle school), sex, age, and factors contributing to infection-related anxiety and educational anxiety that arose from the pandemic. The levels of anxiety and the factors contributing to anxiety were assessed using a 5-point Likert scale ranging from 1 (not at all) to 5 (very anxious).

**Results:**

In an analysis of the data of 237 participants excluding the missing data, many teachers reported feeling infection- and education-related anxiety. A majority of the participants were women (*n* = 152, 64.1%), and the mean age of the participants was 39.8 ± 11.3 years. A stepwise multiple regression analysis identified six factors for infection-related anxiety as significant (*R*^2^ = 0.61, *p* < 0.001). Among these variables, the largest partial regression coefficient value was reported for the following reason: “I feel anxious because we cannot ensure the safety of teachers themselves or of their families” (β = 0.37, *p* < 0.001). For educational anxiety, four of six reasons were identified as significant (*R*^2^ = 0.64, *p* < 0.001). Among these, “anxiety about the students’ home situations” (β = 0.41, *p* < 0.001) and “delay in education (students’ side)” (β = 0.27, *p <* 0.001) had stronger associations with anxiety compared to the others.

**Conclusion:**

In-person education during the COVID-19 pandemic has caused teachers to experience anxiety. This report provides useful information by highlighting the reasons for infection-related anxiety and educational anxiety that teachers experience in face-to-face classes during a pandemic. Even if the coverage of a COVID-19 vaccine becomes widespread worldwide, we will still be combating COVID-19 infections for at least a few years. Given concerns regarding such infections, to ensure students’ right to education, it is essential to understand why teachers feel anxious and to determine appropriate measures to decrease such anxiety.

## Background

Coronavirus infections can be fatal and are a global concern [1]. According to data from United Nations Educational, Scientific and Cultural Organization (UNESCO), as of December 1, 2020, the schools of approximately one in five children around the world, or around 320 million children, were closed [2]. This number has increased by nearly 90 million from 232 million on November 1, 2020, and has continued to increase sharply since October. Because of this increase, UNESCO has disseminated information on school closures around the world, noting that such closures are increasing rapidly worldwide. In addition, UNESCO mentions the magnitude of the impact of school closures on school-aged children and calls on countries to prioritize school reopening and to take all possible actions to make schools safer. UNESCO also notes that the reopening of schools is at the top of the agenda of governments and ministries of education around the globe [3].

As of January 1, 2021, more than 80 million people have been infected with the novel coronavirus (COVID-19), and people around the world are concerned about the risk of infection [4]. Accordingly, school boards and teachers, who typically work in high-risk congregate settings, are taking actions to prevent infections, such as adopting online teaching methods to replace traditional face-to-face classroom environments [5–7]. However, since the onset of the COVID-19 pandemic, adults and children alike have reported experiencing anxiety-induced depression, post-traumatic stress disorder, and other psychological symptoms [8–10]. The impacts of online lessons on children include poor mental health and widening learning disparities among children in low-income families.

Specifically, more students are experiencing negative emotions [11], and the incidence of symptoms, such as anxiety (24.9%), depression (19.7%), and stress (15.2%), have dramatically increased during COVID-19 school closures [12]. Children are vulnerable to psychological distress because their emotional development is not yet stable [13]. School closures interfere with exercise, play, interactions, and communication among students and school friends, directly impacting mental growth, development, and learning [14]. Additionally, children in low-income families often have inferior home learning environments, making it difficult to complete homework and online lessons; thus, the learning gap between children in low- and high-income families is widening [15–18]. Moreover, for many children, schools provide healthy meals that they do not have access to at home, and there is concern that children may not be eating well due to school closures [19, 20].

In Japan, as of May 25, 2020, the state of emergency that was declared for the first time on April 7, 2020, was lifted. Most of the schools that had been temporarily closed in Japan have already reopened as of June 1, 2020. However, the COVID-19 pandemic has not yet subsided. In this context, it is crucial for everyone in the field of education to remain cautious depending on the local situation and to ensure that children’s learning is balanced with the utmost care for infection prevention. Accordingly, the Ministry of Education, Culture, Sports, Science and Technology (MEXT) has taken every possible measure to ensure that children can learn as much as possible without being left behind [21]. According to a survey conducted in Japanese elementary and junior high schools by the MEXT regarding home-based learning during pandemics [21], 100% of schools had students carry out printed textbooks and assignments. In addition, 35% of schools used television broadcasts, and 22% used learning videos produced by the school board for home study. Approximately 35% of schools conducted home study using digital textbooks and digital materials, and 8% offered interactive online instruction between teachers and students. Most schools have been unable to provide interactive online instruction.

Coronavirus infections are known to be less severe in children than in adults [22]. Furthermore, children are unlikely to be virus super-spreaders [23]. However, cases continue to spread throughout Japan, and there have been many outbreaks among primary and middle school students in club activities and classes where infection control was inadequate. Therefore, appropriate measures must be taken to prevent infection while conducting face-to-face classes. Furthermore, to continuously provide children with a fulfilling education, it is necessary to understand the psychological anxiety experienced by teachers holding classes for students during the COVID-19 pandemic as well as the factors thereof.

There have been numerous reports on the psychological effects of COVID-19 [24–28]. In a recent study, factors such as age, sex, teaching level (primary, middle, high school, and university), and school location were related to teachers’ COVID-19-related anxiety levels [29]. However, specific concerns contributing to anxiety levels were not reported. Another study assessed the relationship between mask-wearing and anxiety among teachers in China [30]. Meanwhile, another study examined the prevalence of anxiety disorders in teachers during the COVID-19 pandemic [29]. In any case, the specific reasons behind teachers’ feelings of anxiety concerning COVID-19 have not been investigated. Identifying teachers’ concerns about COVID-19 infection and education during the pandemic will allow us to address the root causes of COVID-19-related anxiety.

The pandemic has also caused serious abuse of human rights, including slander and discrimination [31–33]. Thus, teachers may also be worried about discrimination if they become infected. Moreover, teachers have to educate many students every day. In such situations, teachers can be exposed to various psychological anxieties, such as anxiety about being infected with coronavirus and anxiety about an outbreak in the school. Psychological stress, such as anxiety factors, lead teachers to experience symptoms, such as burnout [34, 35]. Burnout is of particular concern in the teaching profession as it is associated with reduced quality of instruction and diminished ability to effectively engage and teach [36], both of which can lead to potential student harm [37]. Moreover, it affects teacher turnover [38]. Accordingly, understanding the reasons for the teacher’s various anxieties under the COVID-19 pandemic situation and considering the countermeasures against them will ensure that the teacher’s mental health is sound, while maintaining the quality of education for students.

There are some restrictions on school education to avoid COVID 19 infections. If students in their class become infected, the class must be temporarily closed, and students must take a leave of absence from school until they are completely cured [39]. Considering such situations, delay in students’ education is also a matter of concern. For example, there are concerns regarding differences in terms of the learning progress between students who can take face-to-face classes and those who are absent and do their homework at home. Moreover, the study time of some students has reportedly decreased by more than five hours per week owing to the pandemic [40]. Consequently, more research that focuses on the impact of the pandemic on mental health in the field of education is required [41].

Accordingly, we conducted this cross-sectional research study with teachers of primary and middle schools to understand the factors contributing to infection-related anxiety and educational anxiety surrounding the COVID-19 pandemic. The results suggest various factors contribute to anxiety among primary and middle school teachers when resuming school. Moreover, these results can be widely applied as useful information for teachers conducting continuous face-to-face classes even with the ever-present risk of infection.

## Method

### Study design and setting

The present study follows the guidelines of the Strengthening the Reporting of Observational Studies in Epidemiology (STROBE) Statement checklist of items that should be included in reports of cross-sectional studies [42]. Data collection was conducted within 20 days (October 10–30, 2020). The questionnaire was distributed to and collected from teachers at 10 primary schools and 8 middle schools through postal mail in the Shinagawa area of Tokyo. The participants anonymously completed the questionnaires for the collection of demographic data and responses to questions about infection-related anxiety and educational anxiety with respect to the COVID-19 pandemic. When conducting the questionnaire, we distributed explanatory materials to the participants explaining the significance of the survey, which contained the following information: “The purpose is to investigate how teachers are concerned about infection and education after the school is reopened compared to before the school was closed due to the COVID-19 pandemic.” Answers were collected from those who agreed to participate after reading the materials. The survey was conducted at randomly selected schools in the Shinagawa area, Tokyo. Among 397 teachers at the selected schools, 263 participated in the survey (response rate 66.2%) and 237 (response rate 59.7%) responded to all items in the questionnaire and were included in the analysis. All participants were anonymous volunteers.

### Content of the survey instrument

The survey instrument comprised 23 close-ended questions and took approximately 10 min to complete. The 23-item questionnaire was divided into three parts: participant characteristics (3 items: school type, sex, age), factors of anxiety related to infection (13 items/5-point Likert scale: 1 [not at all] to 5 [very anxious]), and factors of anxiety related to education (7 items/5-point Likert scale: 1 [not at all] to 5 [very anxious]). For the question items regarding infectious-related and educational anxiety, we used the results of a survey on the factors of anxiety conducted by Japanese educational magazines on the Internet for teachers (310 respondents: 276 elementary school teachers, 24 junior high school teachers, 5 high school teachers, 7 others) from April 21 to May 6, 2020 [43]. In the aforementioned survey, teachers at currently closed schools were asked what issues they were worried about concerning the school reopening. The infection-related anxiety factors included the lack of vaccines, personal and family security, and students contracting the infection. Education-related anxiety factors included education delays, student education, paucity of time to teach students, dealing with students’ parents, etc. We used the results to create a survey to evaluate the relative strength of anxiety factors.

### Ethical approval

This study was reviewed and approved by the Institutional Review Board Committee of Hoshi University (Approved No. 2020–05). Furthermore, an informed consent form that stated that participation in the study was fully voluntary and that the participants could withdraw from the survey questionnaire at any point was included on the first page of the questionnaire. Informed consent was obtained from all participants.

### Statistical analysis

The obtained data were coded, validated, and analyzed using the free statistical software of R version 3.4.4 (03-15-2018). The questionnaire was completed by 263 teachers, but 26 of them had missing values. Therefore, the data from 237 teachers who answered all items were used for the analysis. The participants’ scores for the factors of their infection-related anxiety and educational anxiety were then calculated as follows: responses of “not at all” received 1 point, while responses of “very anxious” received 5 points each. Count data were expressed in terms of the frequency and percentage; measurement data were expressed as the mean ± SD. A multiple regression analysis employing the stepwise method was used to investigate the factors of infection-related anxiety and educational anxiety. The stepwise multiple regression analysis was performed using infection-related anxiety and g educational anxiety as dependent variables, with the reasons for each item used as independent variables. *P* values of less than 0.05 were considered statistically significant.

## Results

### Demographic characteristics

Table 1 shows the demographic characteristics of the teachers. Of the participants, 64.1% were women, and 34.5% of all the participants were aged 30–39 years. The mean age of the participants was 39.8 ± 11.3 years. Furthermore, 55.7% of the participants were primary school teachers.

**Table 1:**
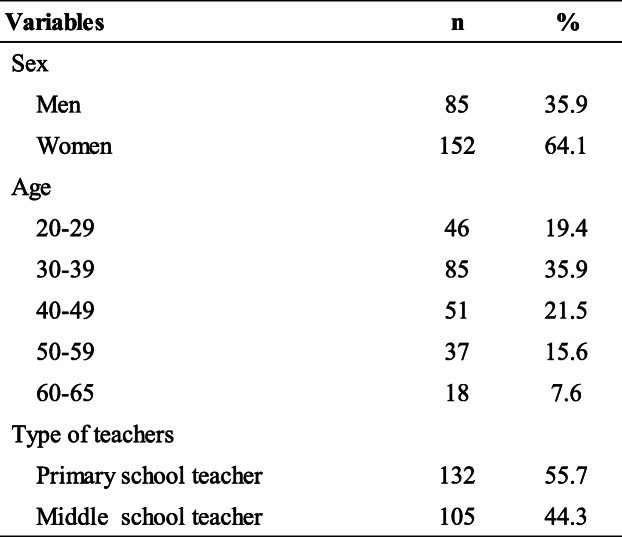
Participant characteristics (*N* = 237)

### Teachers’ infection-related anxiety and educational anxiety owing to the COVID-19 pandemic

**Fig. 1:**
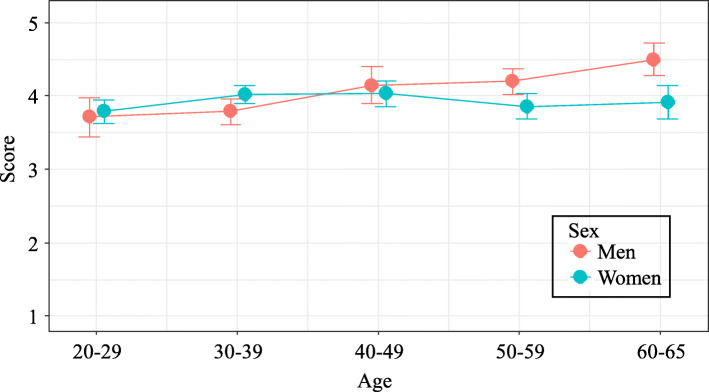
Teachers’ infection-related anxiety because of the COVID-19 pandemic. This figure shows the score for infection-related anxiety (plot points and error bars show the mean score and SE) as per a 5-point Likert scale: 1 (not at all) to 5 (very anxious). Teachers felt infection-related anxiety across all age groups. Furthermore, this anxiety tended to increase with an increase in age for men

**Fig. 2:**
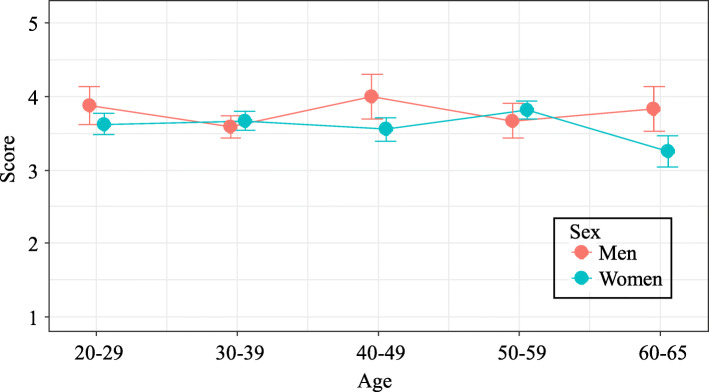
Teachers’ educational anxiety because of the COVID-19 pandemic. This figure shows educational anxiety score (plot points and error bars show the mean score and SE) in a 5-point Likert scale: 1 (not at all) to 5 (very anxious). Educational anxiety was experienced by teachers across all age groups. There were no clear trends for the data concerning educational anxiety with respect to age or sex

Figures 1 and 2 illustrate the participants’ responses regarding infection-related anxiety and education-related anxiety that arose because of the COVID-19 pandemic. The points gained for each item were summed to provide a total score, with a higher score denoting a more negative perception regarding the pandemic (range: 1–5). Overall, infection-related anxiety had an average score of 3.95 ± 0.84. Men tended to be more anxious about infection at older ages (Fig. 1).

Further, educational anxiety had an average score of 3.66 ± 0.81. There were no clear trends for the data concerning educational anxiety with respect to age or sex (Fig. 2).

### Teachers’ scores for the factors of infection-related anxiety and educational anxiety owing to COVID-19

Table 2 illustrates the participants’ responses regarding the factors of their infection-related and educational anxiety because of COVID-19. The results show the scores for 12 questions about infection and 6 questions about education. For anxiety related to infection, the reason “I feel anxious because I don’t know how long it will last” had the highest score (4.53 ± 0.57). For anxiety related to education, the reason “I feel anxious that the physical strength of the child may be reduced” had the highest score (3.97 ± 0.81).

**Table 2:**
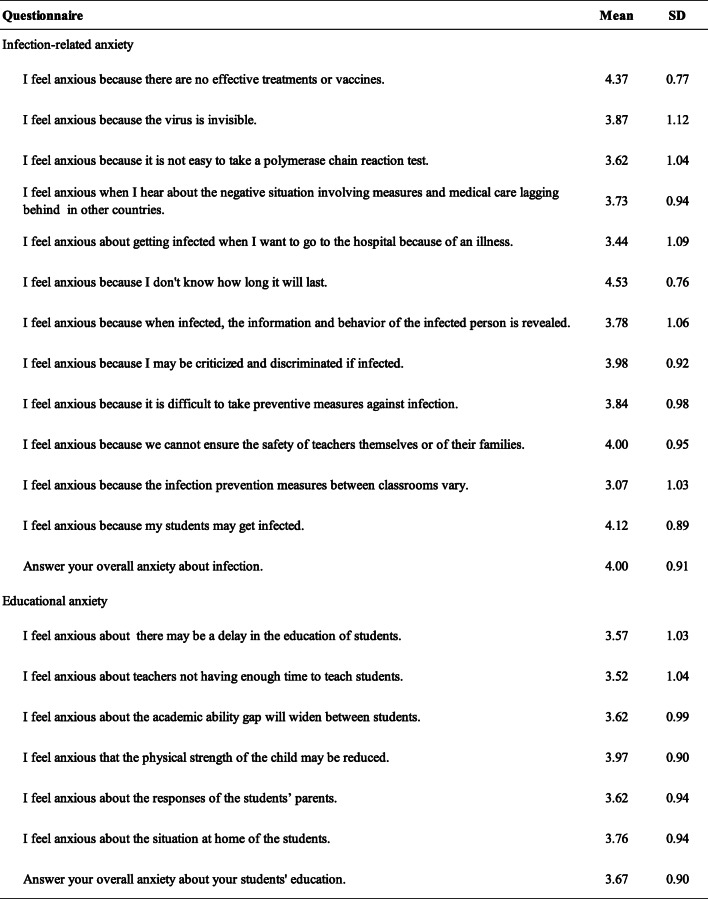
Questionnaire scores for the causes of infection-related and educational anxiety owing to the COVID-19 pandemic

### Factors of infection-related anxiety and educational anxiety in a stepwise multiple regression analysis

Table 3 shows the results of a stepwise multiple regression analysis using the overall infection-related anxiety as the dependent variable and the cause of the infection-related anxiety as the independent variable. Six factors of infection-related anxiety were selected as significant variables (adjusted R^2^) = 0.61, *p <* 0.001). Age, sex, and the type of school were used as confounding variables and thus were not selected as significant variables. The item “I feel anxious because we cannot ensure the safety of teachers themselves or of their families” was the most influential cause (β = 0.37, *p* < 0.001).

**Table 3:**
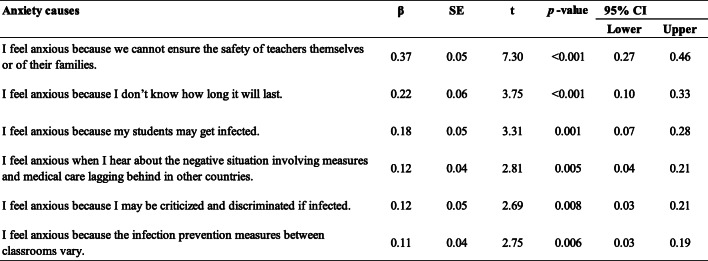
Factors of infection-related anxiety identified by a stepwise multiple regression

Similarly, Table 4 shows the results of a stepwise multiple regression analysis using the overall educational anxiety as the dependent variable and the cause of the educational anxiety as the independent variable. Age, sex, and the type of school were used as confounding variables and thus were not selected as significant variables. Four factors of educational anxiety were selected as significant variables (adjusted *R*^2^ = 0.64, *p* < 0.001). Among them, the items “I feel anxious about the students’ home situations” (β = 0.41, *p* < 0.001)) and “I feel anxious that there may be a delay in the education of students” (β = 0.27, *p* < 0.001) had a stronger association with anxiety than the other items.

**Table 4:**
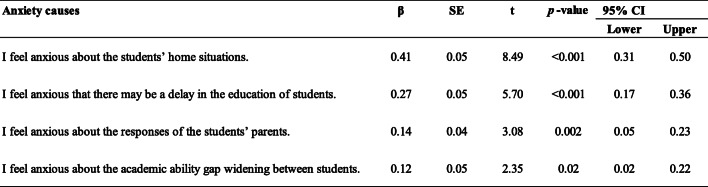
Factors of educational anxiety identified by a stepwise multiple regression

## Discussion

This study provides information on the infection-related anxiety and educational anxiety felt by primary and middle school teachers during the COVID-19 pandemic and the factors thereof. Infection-related anxiety tended to increase with an increase in age for men. Older men are at a higher risk of death from coronavirus infections [44, 45], and the results obtained herein may be related to this information. Furthermore, based on the multiple regression analysis using the stepwise method, six items were extracted as significant factors of infection-related anxiety and four were extracted as significant factors of educational anxiety. The present study shows the factors of infection-related anxiety and educational anxiety. In particular, this study identified the factors of such anxiety after the reopening of schools in the presence of coronavirus infections. Among the identified factors, the safety of the teachers themselves or their families showed higher standardized partial regression coefficient values than other factors (Table 4). In Japan, teachers hold one-hour face-to-face lessons multiple times a day for 30–40 students per class. Consequently, teachers interact with many students daily for a long time, and the risk of infection is high, which may be responsible for the high level of anxiety, among other reasons. Moreover, although the younger generation is less likely to become seriously ill if infected, the risk of student’s getting an infection was associated with anxiety for teachers.

In addition, regarding infection-related anxiety, anxiety concerning the possibility of infection in children and anxiety due to different infection prevention measures between classrooms were identified. Thus, it seems that merely notifying teachers of the guidelines from the national and local governments is insufficient. To provide unified knowledge about and ensure consistent approaches toward infection control, public health professionals, such as doctors and pharmacists, must provide the appropriate lectures and guidance on infection control to teachers and students. Regarding the significant item, “I feel anxious because I don’t know how long it will last,” there was no prospect of convergence at the time of the questionnaire survey; thus, there was concern that education might have to be adjusted for several years in the wake the coronavirus infection. The reason that “knowing the dire situation because countermeasures and medical care cannot catch up with other countries” was a significant factor related to the concern that even if the pandemic temporarily subsides in Japan, it could continue to spread across the world. This was discussed in the daily news, and there were concerns that COVID-19 outbreaks may increase in winter. In fact, many educators are concerned about the potential for two viral infections, influenza and coronavirus, to concurrently affect schools in the winter. Accordingly, these factors may have contributed to anxiety.

The item “I feel anxious because I may be criticized and discriminated if infected” may have been significant for the following reason. In Japan, if a coronavirus infection occurs at school, the school will be temporarily closed. This may increase the anxiety of residents around the school. Furthermore, infections in schools are reported in the media as domestic news, which may have a considerable psychological effect on teachers. To ensure learning opportunities and continue providing education for students, it is essential to properly communicate to the public the fact that teachers are concerned about discrimination and criticism.

Regarding educational anxiety, four items were identified as significant: (1) “I feel anxious about the students’ home situations,” (2) “I feel anxious that there may be a delay in the education of students,” (3) “I feel anxious about the responses of the students’ parents,” and (4) “I feel anxious about the widening gap in academic ability among students.” In particular, items (1) and (2) had high standard partial regression coefficients compared to the other factors, which may be because students spend more time at home owing to the implementation of infection control measures such as refraining from club activities and shortening class hours. In nuclear families where parents work during the day, it is believed that students spend more time at home alone during the day, thereby increasing teachers’ anxiety. Regarding “anxiety about dealing with parents,” many schools have temporarily shut down parent associations due to the coronavirus. Teacher-parent communication has also changed. Even if schools reopen, it is likely that extracurricular activities, such as athletic meetings, will be canceled. As a result, parents are worried about students’ school life and have become increasingly demanding toward the schools and teachers. With regard to “concerns about widening academic disparities,” wealthy families can enhance their children’s at-home education via tutoring, online learning on computers and tablets, and distance learning. However, some families are unable to access such services and tools, thereby increasing academic disparities.

Many studies prior to the pandemic found that online lessons are useful because they offer an extremely efficient and diverse range of electives [46]. However, some reports found that it is difficult to conduct online classes for those that involve physical activity [9] and that providing mental care for students is challenging [47]. Therefore, even if the COVID-19 pandemic subsides, face-to-face lessons will be required. It will then be necessary to understand—in detail—the factors of teachers’ anxiety reported herein and to strive to provide an environment that eliminates anxiety as much as possible.

### Limitations

The present study has several limitations. First, this study has a cross-sectional design and was carried out during 2 weeks of autumn in which there were relatively few cases of coronavirus infections in Japan; thus, many possible problems may not have emerged during the course of the research. Constant attention and further investigation are required to assess the overall situation. Second, this survey was administered to school teachers in Tokyo, and it does not reflect the opinions of teachers in other areas. The density of contact with people differs between large cities and rural areas; accordingly, the situation in terms of the possibility of infection also differs between these contexts. In Japan, the capital Tokyo has the largest population and the highest risk of infection. Conversely, the possibility of infection is not as high in the countryside. Third, the educational environment, such as the number of students and facilities, varies depending on the country. Different countries have different infection control methods, and there may be different reasons for teachers’ anxiety. Therefore, it is necessary to conduct similar surveys and obtain the results thereof in other countries. Furthermore, another limitation of this study was the lack of demographic information on the teachers who completed the questionnaires. Regarding the male–female ratio of participants, 68% of the participants identified as female. The percentages of female teachers in elementary and junior high schools in Japan are approximately 63 and 44%, respectively [48]. The percentage of elementary school teachers who participated in our study was high, which may have led to a high percentage of female teachers. Therefore, the male–female ratio of the teachers in each country must be considered. Moreover, the study did not use a validated questionnaire; therefore, the results should be carefully interpreted. Nevertheless, this study provides valuable information to address the concrete causes of teachers’ anxiety following the reopening of schools in the presence of COVID-19. In this study, we searched for factors associated with anxiety by variable selection using the stepwise method. Among the selected factors, we then demonstrated the strength of the factors associated with anxiety. These findings provide important information to formulate measures to eliminate infection- and education-related anxiety factors.

The psychological wellbeing of teachers who are in a position to guide students is important to ensure the students’ right to education. The results of this study are useful not only for providing information regarding the anxiety of Japanese teachers but also for providing information that can be helpful when other countries are considering reopening schools.

## Conclusions

In-person education during the COVID-19 pandemic has caused teachers to experience anxiety regarding infection and education. Although vaccines are starting to be implemented in various countries, it is unlikely that the virus will be completely eliminated. The study is expected to be used as information on the factors of teachers’ infection-related and educational anxiety when resuming school in the presence of coronavirus infections. From the results obtained, it could be insufficient to notify teachers of the guidelines from the national and local governments. Local public health professionals, such as doctors and pharmacists, must provide teachers with knowledge, attitudes, and practice regarding infectious diseases to eliminate teachers’ infection and educational anxiety.

## Data Availability

Data is available from the corresponding author upon reasonable request.

## Abbreviations

CI: Confidence interval
COVID-19: Coronavirus disease, 2019
SD: Standard deviation
SE: Standard error
